# Effects of Cell Phone Dependence on Mental Health Among College Students During the Pandemic of COVID-19: A Cross-Sectional Survey of a Medical University in Shanghai

**DOI:** 10.3389/fpsyg.2022.920899

**Published:** 2022-06-27

**Authors:** Ting Xu, Xiaoting Sun, Ping Jiang, Minjie Chen, Yan Yue, Enhong Dong

**Affiliations:** ^1^School of Nursing and Health Management, Shanghai University of Medicine and Health Science, Shanghai, China; ^2^School of Media and Communication, Shanghai Jiao Tong University, Shanghai, China; ^3^Institute of Health Yangtze River Delta, Shanghai Jiao Tong University, Shanghai, China; ^4^Shanghai Tenth People's Hospital, Tongji University School of Medicine, Shanghai, China; ^5^Genetics and Biochemistry Laboratory, Shanghai Mental Health Center, Shanghai, China; ^6^Renji Hospital, Shanghai Jiao Tong University School of Medicine, Shanghai, China; ^7^School of Health Care Management, Tsinghua University, Beijing, China

**Keywords:** cell phone dependence, mental health, effect, college students, China

## Abstract

**Objective:**

To investigate the effects of cell phone dependence (CPD) on mental health among undergraduates during the COVID-19 pandemic and further identify the determinants that may affect their mental health in China.

**Methods:**

The data were collected from 602 students at a medical school in Shanghai *via* an online survey conducted from December 2021 to February 2022. The Mobile Phone Addiction Index (MPAI) and Depression Anxiety Stress Scale (DASS) were applied to evaluate CPD and mental health, respectively. Independent sample *t*-test and one-way analysis of variance (ANOVA) were employed to compare the means of continuous variables among categorical groups. Correlations between continuous variables were detected using Pearson's correlation analysis. Univariable and multivariable logistic regressions were employed to identify the determinants of mental health.

**Results:**

Among the 402 eligible students, 73.88% were women with an average age of 20.19 ± 2.36 years. On average, the DASS score was 32.20 ± 11.07, the CPD score was 36.23 ± 11.89, and the cell phone use duration was 7.67 ± 3.61 h/day. CPD was found to have a negative effect on mental health among college students in Shanghai. Additionally, cell phone use duration, age, being senior students, faculty-student relationship, insomnia, tobacco use, obesity, and life satisfaction were clarified as contributing factors to mental health among college students.

**Conclusion:**

High degree of CPD could have a negative effect on college students' mental health, which might lead to some psychological problems. Appropriate actions and effective interventions are highly needed to prevent severe psychological injuries among college students in China.

## Introduction

Coronavirus Disease-19 has become a global public health problem since its outbreak in early 2020. It not only causes a number of physical problems but also affects people's mental health. Currently, the COVID-19 pandemic still continues to challenge people's livelihoods and economies around the world and bring tremendous changes to people's daily life (Moreno et al., 2020; Vindegaard, 2020; Kooli, 2021). The education industry, especially the higher education industry is one of the most affected fields. Due to the prevention and control of COVID-19 requirements, such as online teaching, outdoor activities' suspension, and gatherings prohibition in the public field, college students have to increase their online time and social media usage, which result in poor sleep quality, irregular eating pattern, and even anxiety and depression (Fu et al., 2021). As the outbreak of COVID-19, the governments had initiated the appropriate program of health education and relevant regulations to strictly prevent the spread of COVID-19, such as outdoor activities' suspension, social distance keeping, and gatherings prohibition in the public field. For college students, they had to be required to come to home study online or in group isolation in school dormitories, which increased their online time, social media usage, and potentially changed their learning and life styles. Despite cell phones could bring convenience to people, inappropriate usage of mobile devices has the potential to be detrimental. For example, many people used cell phones frequently, leading to internet addiction (Ozturk, 2021). One study showed more frequent cell phone overuse among college students when compared with the pre-epidemic period (Kim, 2018). Additionally, it was also reported that frequent use of the internet on cell phones among college students would cause poor sleep quality, irregular eating patterns, and even anxiety and depression (Liu et al., 2021). Within the Chinese context, according to a report from the China Internet Network Information Center (CINIC), up to December 2020, there were 1.614 billion mobile phone users in China, among whom students accounted for 21%, with college students aged 18–22 being the largest- and fastest-growing group (China Internet Network Information Center, 2021; Conrad et al., 2021). Especially in the era of routine epidemic control and prevention after 2020, college students are used to employ mobile phones as their daily life instrument for conducting interactive learning activities, such as taking online lessons and completing class assignments (Shi et al., 2021). However, there still without exception existed cell phone dependence (CPD) among Chinese college students (Hong et al., 2021). CPD is described as the compulsive habit of avoiding reality or producing excitement *via* cell phone use with symptoms, such as salience and withdrawal (Lam et al., 2009). It included any behavioral addiction thought to be similar to that of an internet, gambling, shopping, or video game addiction (Chen and Oliffe, 2018). Though no evidence was present to show that the COVID-19 pandemic directly altered the relationship between CPD and mental health, some studies had found the mediating and moderating role of cyberchondria severity in the association between fear of COVID-19 and smartphone addiction among individuals (Kayis et al., 2021). Literature has reported that CPD was associated with social and emotional intelligence (Zou et al., 2019a), in which it demonstrated that the lower emotional intelligence (or lack it entirely) one has, severer CPD is (Xie et al., 2019a). Excessive CPD was also found to be associated with difficulties in cognitive-emotion regulation, impulsivity, impaired cognitive function, addiction to social networking, shyness, and low self-esteem (Volkmer, 2018; Oviedo-Trespalacios et al., 2019). For college students, it is also reported that CPD may have a negative impact on them in terms of time allocation and management, school performance, interpersonal relationships, and health (Dempsey et al., 2020).

College students have high need of accessing knowledge through digital platforms because they often need to search for extra information during their learning activities and clinical practices. They are also required to gain skills of delivering health services through mobile platforms, due to the fast development of mobile health (mHealth) and electronic health (eHealth). However, current literature lacks in-depth research on how CPD may affect college students on their health and performances. Therefore, the purpose of this study was to investigate the effects of CPD on mental health among undergraduates in Shanghai, China during the COVID-19 pandemic.

In order to identify independent effects of CPD on mental health for college students, other related characteristics must be controlled. We take into account age (Xie et al., 2019b), grade (Dou et al., 2020), gender (Lu et al., 2018), monthly allowance (Li et al., 2020), Faculty-Student relationship (Liang and Liu, 2021), substance use (Carreiro et al., 2018), physical exercise (Zhong and Wang, 2020), insomnia (Zhang et al., 2021), obesity (Ali et al., 2021), life satisfaction (Kuang-Tsan, 2017), and subjective wellbeing (SWB) (Ye et al., 2021) to confirm the salient effect of CPD and mental health.

## Materials and Methods

### Data Sources

Firstly, to ensure the representativeness of the sample, we used the following formula to calculate the required minimum sample size according to Krejcie and Morgan (1970):


(1) $$\begin{array}{r} n=\frac{Z_{1-\alpha }^{2}NP\left(1-P\right)}{d^{2}\left(N-1\right)+Z_{1-\alpha }^{2}P\left(1-P\right)} \end{array}$$

Where n refers to the required minimum sample size, Z_1−α_ is the value from the standard normal distribution for the selected confidence level (e.g., for α = 0.05, for 95% confidence level, and Z = 1.96). N is the given population size. P refers to the prevalence of the interest; and d is the degree of accuracy. In this study, we set P to 0.228 (Zou et al., 2019b), α is 0.05, and d is 0.05. The value of N is 10,000, so we get the required minimum size equal to 263. Considering the follow-up loss rate, rejection rate, and questionnaire efficiency, we finally set the needed minimum sample size as 600.

The data were collected from a convenience sample of 602 students at a medical school in Shanghai using an online survey with a total of 80 questions from December 2021 to February 2022. The online questionnaire was set to be answered through a single IP address. If the survey was detected to be (1) completed within <200 s (61, 10.13%); (2) provided with repeated answers or a certain pattern of answers (98, 16.28%); (3) having 5% or more missing values (20, 3.33%); or (4) having logical errors (21, 3.49%), it will be classified as unqualified and excluded. After exclusion, 402 eligible participants were enrolled in the final analyses. The relatively low response rate (66.77%) was due to the abovementioned rigid exclusion criteria, which made many questionnaires excluded for quality reasons.

To reduce the privacy concern, the respondents did not need to fill in their real names, contact information, or other private information. The questionnaire designed by the research group contained the following variables: (1) demographic and socioeconomic characteristics (e.g., age, gender, ethnicity, grade, major, monthly expenditure, etc.), and (2) seven questions on mental health status, CPD, substance abuse, faculty-student relationship, physical activity, body mass index (BMI), life satisfaction, and SWB.

### Measurements

Mental health was measured using the Chinese version of the Depression Anxiety Stress Scale (DASS) (Zhang et al., 2021), a 21-item scale that includes three dimensions of mental health—depression, anxiety, and stress. Each item was anchored from “Don't apply to me at all” (scored with 1) to “Applied to me very much” (scored with 4), with higher scores indicating severer psychological problems. In the present study, all three dimensions and the total scale had good reliability and validity. The internal consistency coefficients of the three subscales were 0.917, 0.878, and 0.899, respectively. The internal consistency coefficient of the total scale was 0.961.

Cell phone dependence was measured using the Chinese version of Mobile Phone Addiction Index (MPAI) (Liu et al., 2021), a 17-item scale that includes four dimensions: inability to control craving, feeling anxious and lost, mood improvement, and productivity loss. The 17 items were answered on a five-point Likert scale with 1 indicating “not at all” and 5 indicating “always.” The total score was obtained by summing up the scores of 17 items. The higher the score of MPAI was, the greater the level of mobile phone addiction was. In this study, all four dimensions and the total scale had good reliability and validity. The internal consistency coefficients of the total scale and the three subscales were 0.912, 0.828, 0.800, 0.891, and 0.815, respectively.

The satisfaction with life scale (SWLS), a five-question scale, was used to measure individual satisfaction with life as a whole (Bieda et al., 2018; Li et al., 2019). Respondents rated the scale on a five-point Likert scale from “very dissatisfied” (assigned a score of 1) to “very satisfied” (assigned a score of 5), with higher scores indicating higher satisfaction with life. In the present study, the internal consistency coefficient of the scale was 0.913.

Subjective wellbeing is defined as a person's evaluative response to his or her life and can be divided into cognitive components, such as life satisfaction and affective components, i.e., happiness. This paper used Stubbe et al.'s SWB scale, which also contains five questions measuring individuals' life satisfaction and happiness (Nie and Ma, 2020; Zhang et al., 2020). The respondents rated the scale on a Likert scale from “strongly disagree” (assigned a score of 1) to “strongly agree” (assigned a score of 5), with higher scores indicating higher SWB. In the present study, the internal consistency coefficient of the scale was 0.894.

Faculty-student relationship was measured using the Student-Faculty Relationship Perception Questionnaire developed by Yu et al. (2017), which contains seven items. It was adopted from the “Leader-member exchange differentiation” questionnaire (Yu et al., 2017; Liang and Liu, 2021), which is a widely used relationship measurement tool based on social exchange theory (Khuram et al., 2021). The internal consistency coefficient of this measurement in this study was 0.945.

The measurement and type for the other key variables can be seen in Appendix 1.

### Date Analysis

In the first step, an S-K test was conducted to test the normality of the data and followed by a descriptive analysis of the data, such as demographic and socioeconomic characteristics, faculty-student relationships, physical activity, substance abuse, CPD, life satisfaction, and SWB. Then, we conducted an independent sample *t*-test and one-way analysis of variance (ANOVA) to determine whether there were any statistically significant differences in the means of two and more independent groups. Additionally, Pearson's correlation analysis was performed to test linear relationships between continuous variables. Lastly, we conducted univariate (model 1—unadjusted model) and multivariable logistic regressions to calculate the effects of the CPD on mental health. Variables that were significant in univariate analyses at a liberal *p*-value (*p*-value < 0.5) were retained as covariates for the adjusted models (models 2 and 3). Model 2 controlled for the variables, such as personal characteristics, faculty-student relationship, insomnia, substance abuse, and physical exercise, while model 3 additionally added life satisfaction and SWB. To check for multi-collinearity in the independent variables, we used the Variance Inflation Factor (VIF) technique. The variable is acceptable and will be included in regression analysis if its VIF is <5.

All analyses were conducted using Stata.15.0 (StataCorp LP, College Station, TX, USA). Statistical significance of the results was based on *p* < 0.05 two-tailed test.

## Results

### Descriptive Characteristics

From a total of 602 returned surveys, 402 respondents (66.7%) were selected according to the exclusion criteria [297 women (73.88%), 105 men (26.12%)], among which the majority of Han nationality (*n* = 368) accounted for 91.54%. The average age of the students was 20.19 ± 2.36, 127 (31.59%) were sophomores, 283 (70.40%) were unmarried, and 307 (76.37%) were living in city hometowns. The mean DASS score was 32.20 ± 11.07, the mean CPD score was 36.23 ± 11.89, the cell phone use duration was 7.67 ± 3.61 h/day, the faculty-student relationship score was 3.80 ± 0.81, the life-satisfaction score was 18.7 ± 4.15, and the SWB score was 15.90 ± 1.47. The other variables are detailed in Table 1.

**Table 1:** Characteristics of participants (*N* = 402).

**Characteristic**	***N***	**%**
**Gender**		
Male	105	26.12
Female	297	73.88
**Grade**		
Freshman	107	26.62
Sophomore	127	31.59
Junior	120	29.85
Senior	48	11.95
**Hometown type**		
Urban	307	76.37
Rural	95	23.63
**Ethnic group**		
Han	368	91.54
Minority nationality	34	8.46
**Specialty**		
Medical	235	58.46
Health economy and management	167	41.54
**Relationship status**		
Not dating nor married	283	70.40
Dating but unmarried	108	26.87
Married	9	2.24
Others	2	0.50
**Monthly allowances (RMB)**		
<1,000	24	5.97
1,000–1,499	143	35.57
1,500–1,999	137	34.08
2,000–2,499	59	14.68
2,500–2,999	19	4.73
>3,000	20	4.98
**Insomnia**		
No	162	40.30
Seldom	153	38.06
Sometimes	63	15.67
Often	19	4.73
Daily	5	1.24
**Physical activity**		
Never	23	5.72
Rare (≤ 2 times/month)	148	36.82
Sometimes (1–2 times/month)	182	45.27
Often (3–5 times/month)	41	10.20
Daily	8	1.99
**Cigarette use**		
Never	380	94.53
Ex-smoker	8	1.99
Current smoker	14	3.48
**Alcohol use**		
Never	260	64.68
Rare (≤ 2 times/month)	103	25.62
Sometimes (≤ 4 times/month)	29	7.21
Often (≤ 12 times/month)	5	1.24
Always (>12 times/month)	5	1.24
**BMI index**		
Low weight	66	16.42
Normal	250	62.19
Overweight	57	14.18
Obesity	29	7.21
	**Mean**	**SD**
**Age (year)**	20.19	2.36
**Phone use duration (hours)**	7.67	3.61
**DASS**	32.20	11.07
**MPAI**	36.23	11.89
**SWLS**	18.71	4.15
**SWB**	15.90	1.47
**F-S relationship**	3.80	0.81

*DASS, Depression Anxiety Stress Scales; MPAI, mobile phone addiction index; SWLS, The satisfaction with life scale; SWB, Subjective well-being; F-S relationship, Faculty-Student relationship*.

### Univariate Analyses and Correlation Analyses

Through the S-K normality test, the data of all the dependent and independent variables of interest for this study were acceptable to conduct the following analysis, with the skewness values being all near zero, and the kurtosis values being all <3. Moreover, the means of CPD were significantly different between the two gender groups (*t* = −1.91, *p* = 0.0286). The means of CPD were also different between at least two of the insomnia groups (*F* = 3.96, *p* = 0.004), physical exercise groups (*F* = 4.80, *p* = 0.001), alcohol use groups (*F* = 2.41, *p* = 0.049), and BMI groups (*F* = 5.85, *p* = 0.047). In addition, the variables that were found to be significantly associated with CPD included the cell phone use duration (*r* = 0.249, *p* < 0.001), faculty-student relationship (*r* = −0.200, *p* < 0.001), and life satisfaction (*r* = −0.194, *p* < 0.001). Similarly, the variables significantly associated with DASS were CPD use duration (*r* = 0.259, *p* < 0.001), faculty-student relationship (*r* = −0.085, *p* = 0.0471), life satisfaction (*r* = −0.209, *p* < 0.001), and SWB (*r* = −0.219, *p* < 0.001). The mean of DASS was significantly different between at least two of the three or more groups when classifying by grade (*F* = 4.64, *p* = 0.003), monthly allowance (*F* = 2.24, *p* = 0.049), insomnia (*F* = 12.26, *p* < 0.001), physical exercise (*F* = 2.77, *p* = 0.027), tobacco use (*F* = 3.61, *p* = 0.028), alcohol use (*F* = 3.50, *p* = 0.008), and BMI (*F* = 5.41, *p* = 0.046). Details of the above analyses are shown in Table 2.

**Table 2:** Univariate/correlation analysis results (*N* = 402).

**Variable**	**CPD score**			**DASS score**		
	**Mean ±SD**	***t/F-Value***	***P***	**Mean ±SD**	***t/F-Value***	***P***
**Age (year)**		0.027	0.583		−0.037	0.455
**Phone use duration (hours)**		0.249^a^,***	<0.001		0.259***	<0.001
**Grade**		1.09	0.3518		4.64	0.003
Freshman	34.93 ± 12.07			30.47 ± 10.91		
Sophomore	35.80 ± 11.04			32.44 ± 9.79		
Junior	37.07 ± 12.66			31.45 ± 11.16		
Senior	39.12 ± 10.65			38.70 ± 12.54		
**Gender**		−1.9076*	0.029		0.6337	0.527
Male	34.33 ± 12.42			32.84 ± 12.37		
Female	36.90 ± 11.64			31.98 ± 10.58		
**Ethnic groups**		−1.5900	0.056		−1.167	0.244
Han	35.94 ± 11.83			32.01 ± 10.95		
Minority nationality	39.32 ± 12.22			34.32 ± 12.29		
**Hometown type**		−0.6640	0.254		−0.685	0.494
Urban	36.01 ± 12.12			31.99 ± 11.13		
Rural	36.94 ± 11.11			32.88 ± 10.90		
**Relationship status**		1.07	0.360		0.35	0.792
Not dating nor married	35.85 ± 11.61			32.23 ± 11.09		
Dating but unmarried	36.70 ± 12.22			31.81 ± 10.59		
Married	39.78 ± 15.82			34.89 ± 16.73		
Others	48.00 ± 12.73			37.00 ± 7.07		
**Monthly allowances (RMB)**		2.18	0.056		2.24*	0.049
<1,000	35.79 ± 11.42			35.46 ± 12.76		
1,000–1,499	36.45 ± 11.67			32.76 ± 11.44		
1,500–1,999	34.27 ± 11.13			29.97 ± 9.87		
2,000–2,499	38.32 ± 12.74			34.27 ± 11.30		
2,500–2,999	42.37 ± 12.08			34.53 ± 11.54		
>3,000	36.60 ± 14.29			31.30 ± 11.23		
**Specialty**		−0.606	0.272		0.028	0.977
medical	35.93 ± 12.02					
Health economy and management	36.67 + 11.71					
**Faculty-student relationship**		−0.200^a^,***	<0.001		−0.085^a^,*	0.047
**Insomnia**		3.96**	0.004		12.26***	<0.001
NO	34.27 ± 12.10			28.50 ± 9.83		
Seldom	35.99 ± 10.40			32.92 ± 9.93		
Sometimes	40.89 ± 13.11			36.65 ± 12.94		
Often	38.16 ± 12.59			41.37 ± 8.87		
Daily	40.80 ± 15.52			39.40 ± 18.50		
**Cigarette use**		0.63	0.534		3.61*	0.028
Never	36.08 ± 11.88			31.98 ± 10.89		
Ex-smoker	37.13 ± 12.63			29.75 ± 11.49		
Current smoker	39.64 ± 11.83			39.79 ± 13.65		
**Alcohol use**		2.41*	0.049		3.50**	0.008
Never	35.15 ± 11.59			31.04 ± 10.58		
Rare (≤ 2 times/month)	38.32 ± 11.65			33.83 ± 11.13		
Sometimes (≤ 4 times/month)	36.31 ± 13.56			33.52 ± 11.00		
Often (≤ 12 times/month)	47.00 ± 11.77			44.60 ± 16.24		
Always (>12 times/month)	37.80 ± 15.55			39.00 ± 18.62		
**Physical exercise**		4.80**	0.001		2.77*	0.027
Never	42.35 ± 14.50			37.13 ± 14.10		
Rare (≤ 2 times/month)	37.86 ± 12.36			33.57 ± 11.66		
Sometimes (1–2 times/month)	35.21 ± 10.82			30.80 ± 9.85		
Often (3–5 times/month)	33.39 ± 10.64			31.51 ± 10.50		
Daily	26.00 ± 13.18			28.25 ± 14.54		
**BMI**		5.85*	0.047		5.41*	0.046
Low weight	38.61 ± 1.04			32.62 ± 1.34		
Normal	36.37 ± 1.76			31.93 ± 10.41		
Overweight	32.90 ± 12.47			31.51 ± 10.67		
Obesity	36.14 ± 12.73			35.00 ± 15.98		
**SWLS**		−0.194^a^,***	<0.001		−0.209^a^,***	<0.001
**SWB**		−0.070^a^	0.1613		−0.219^a^,***	<0.001

a *Coefficient by Pearson Correlation Analysis*.

* *p < 0.05*.

** *P < 0.01*.

*** *P < 0.001*.

### Logistic Regression Analyses

Through the multi-collinearity test, each VIF of the predictors significant in univariate analysis was included in the logit regression model and was between 1.06 and 2.06, indicating no multicollinearity will occur in the regression analysis.

The unadjusted odds ratio (OR) value of CPD was 1.069 (*p* < 0.001; model 1) (Table 3). Similarly, as seen in model 2, the OR of CPD, cell phone use time, age, senior year, frequent insomnia, current smoking cessation, and obesity are statistically associated with mental health. Among them, the OR of CPD is 1.072 (*p* < 0.001), indicating that for every 1 increase in the CPD score, a college student is 1.07 times as likely to have mental issues.

**Table 3:** Logistic regression analysis results (*N* = 402).

**Variable**	**Model 1 (Unadjusted model)**	**Model 2 (Adjustment model)**	**Model 3 (Adjustment model)**
	**OR**	**OR**	**OR**
**CPD**	1.069*** (1.046–1.093)	1.072*** (1.047–1.098)	1.075*** (0.048–0.097)
**Cell phone use duration**	1.122*** (1.052-1.193)	1.092* (1.009–1.183)	1.090* (1.003–1.185)
**Age**		0.822* (0.701–0.963)	0.845** (0.722–0.989)
**Grade**
Freshman		1.000	1.000
Sophomore		2.336 (0.944–5.781)	2.471 (0.958–6.374)
Junior		2.244 (0.850–5.919)	2.560 (0.941–6.961)
Senior		8.417** (1.922–36.857)	8.019** (1.760–36.54)
**F-S relationship**		0.582** (0.027–0.989)	0.577** (0.029–0.919)
**Insomnia**
No		1.000	1.000
Seldom		1.820 (0.961–3.445)	1.770 (0.924–3.390)
Sometimes		2.079 (0.939–4.604)	1.792 (0.836–3.840)
Often		7.043*** (2.414–20.552)	6.390** (2.083–19.60)
Daily		2.158 (0.045–103.136)	3.347 (0.138–80.88)
**Physical exercise**
Never		1.000	1.000
Rare (≤ 2 times/month)		2.774 (0.909–8.466)	2.787 (0.932–8.332)
Sometimes (1–2 times/month)		2.080 (0.680–6.360)	1.959 (0.657–5.846)
Often (3–5 times/month)		1.558 (0.416–5.837)	1.566 (0.423–5.801)
Daily		1.636 (0.153–17.525)	2.289 (0.192–27.32)
**Cigarette use**
Never		1.000	1.000
Ex-smoker		0.169* (0.030–0.951)	0.0996** (0.0199–0.499)
Current smoker		0.896 (0.157–5.127)	0.829 (0.149–4.618)
**Alcohol use**
Never		1.000	1.000
Rare (≤ 2 times/month)		1.275 (0.658–2.471)	1.270 (0.656–2.459)
Sometimes (≤ 4 times/month)		1.875 (0.729–4.820)	2.642 (0.970–7.195)
Often (≤ 12 times/month)		9.750 (0.352–270.248)	14.90 (0.530–418.4)
Always (>12 times/month)		2.517 (0.205–30.914)	2.429 (0.147–40.13)
**BMI index**
Low weight		1.000	1.000
Normal		1.191 (0.572–2.480)	1.176 (0.572–2.417)
Overweight		1.862 (0.717–4.840)	1.988 (0.774–5.107)
Obesity		3.979** (1.486–10.654)	3.693** (1.360–10.03)
SWLS			0.913** (0.836–0.996)
SWB			0.880 (0.729–1.063)
Constant *t*	0.025 (0.010–0.059)	0.078 (0.003–2.130)	0.555 (0.0114–27.06)
Pseudo *R*^2^-value	0.095	0.198	0.219
Observation	402	402	402

* *p < 0.05*.

** *P < 0.01*.

*** *P < 0.001*.

The OR of cell phone use duration is 1.092 (*p* < 0.05), revealing that the more time students spend on mobile phones, the greater risk of psychological problems will occur, that is, with every 1 h increase in cell phone use time, a college student is 1.09 times as likely to have mental issues. Contrastly, the OR of age is 0.822 (*p* < 0.05), exhibiting for every 1 year increase, a college student is only 82.2% as likely to have mental issues. Similarly, as the OR of the faculty-student relationship is 0.582 (*p* < 0.01), exhibiting that the OR of having severe psychological problems will decrease as a relationship between faculty and student develops. It was the case for the students who have quit smoking as compared to non-smokers.

Similarly, controlling the same confounders, in model 3, we also got the similar results for three sub-dimensions: depression, anxiety, and stress when regressing CPD and cell phone use duration (see Appendix 2).

Senior students relative to freshmen, OR = 8.42, had greater odds of having mental disorder. It was also the case for students having a frequent insomnia (OR = 7.043, *p* < 0.001) and being obesity (OR = 3.979, *p* < 0.01) as compared to their counterparts, respectively.

Similarly, in model 3, when taking consideration of additional life satisfaction and SWB based on the model 2, it can be also seen that CPD (OR = 1.075, *p* < 0.001), cell phone time duration (OR = 1.090, *p* < 0.05), age (OR = 0.845, *p* < 0.01), faculty-student relationship (OR = 0.577, *p* < 0.01), senior-year (OR = 8.019, *p* < 0.01), frequent insomnia (OR = 6.390, *p* < 0.01), current smoking cessation (OR = 0.01, *p* < 0.01), and obesity (OR = 3.693, *p* < 0.01) still have the statistically significant association with mental health. Additionally, life satisfaction also shows a statistically significant effect on mental health. As an illustration, the OR for college students whose life satisfaction is 0.913 (*p* < 0.05), indicating that for every 1 increase in life satisfaction score, a college student is only 91.3% as likely to have psychological issues.

## Discussion

The findings showed that the average time that college students spent on their cell phones was 8 h a day, which was much longer than previous studies during the COVID-19 pandemic (Mach et al., 2020; Jiang et al., 2021).

For example, the average duration of mobile phone use for university students reported by Jiang et al. (2021) in Shanghai was 7.39 h each day during the COVID-19 pandemic. It indicated these students were somewhat addicted to their cell phones (Jiang et al., 2021). After adjusting confounders in the logistic regression analyses, this study supported the significant effects of CPD on decreasing mental health among college students in Shanghai (Zou et al., 2021). Additionally, cell phone use duration was also significantly clarified as contributing factor to mental health among college students (Tao et al., 2016; Liu et al., 2019). These findings would help educational institutions to address the disruptive cell phone addiction behavior among the students (Li, 2021).

The finding that CPD was negatively associated with mental health indicated that college students who have greater CPD were more likely to suffer from serious psychological problems (Lopez-Fernandez et al., 2014; Zhen et al., 2019). It hinted that excessive CPD would increase the experiences of mental health issues, such as depression, anxiety, and tension/stress, for college students. Our study contributed to the existing evidence of CPD that is negatively affecting college students' mental health and also identified a group of other significant determinants of mental health, which should be considered to improve the psychological resilience of college students in China. Interestingly, we also found that there was a positive relationship between the mobile phone use duration and mental health problems, indicating that the prolongation of cell phone use time would directly increase the probability of mental health problems in college students, which was consistent with the findings from previous studies (De-Sola Gutiérrez et al., 2016; Cha, 2018). It hinted that some international guidelines were needed for the integration of psychosocial support and mental health promotion intervention among colleges and universities during the pandemic of COVID-19. Accordingly, college instructors were suggested to allocate time for mental health support to relieve students' concerns and worries through reducing the overuse of cell phones by (1) signing cell phone use agreements in the classroom (Zhong and Wang, 2020), (2) designing innovative experiments and interactive learning activities to attract students to participate in classroom learning (Subba et al., 2013), and (3) guiding college students to engage in hobbies or activities that do not involve their cell phones, such as playing a musical instrument or painting to balance their engagement in the real world along with screen time (Sumuer, 2021).

Other covariates, such as physical exercise, alcohol use, and SWB, had non-significant effects on CPD. In contrast, age, grade, faculty-student relationship, insomnia, cigarette use, BMI, and life satisfaction were significantly associated with mental health. These findings echoed prior observations on the determinants of mental health for college students (Lopez-Fernandez et al., 2014; Fergusson et al., 2015; De-Sola Gutiérrez et al., 2016; Bieda et al., 2018; Cha, 2018; Zhen et al., 2019; Nie and Ma, 2020; China Internet Network Information Center, 2021; Jiang et al., 2021; Liu et al., 2021). Notedly, in this study, having higher life satisfaction was associated with lower odds of suffering from psychological problems for college students, which indicated life satisfaction's role in reducing the psychological problems. These findings were all consistent with Fergusson et al. (2015), Anderssen et al. (2020), and Duong (2021). It can be explained that with the improvement of life satisfaction, college students might have positive and stable attitudes toward their own living conditions, recognize the true value of life, pursue work-leisure balance, and actively conduct coping strategies (such as participating in physical exercise) to relieve the pressure, depression, stress, and anxiety they encountered in college life (Zhai et al., 2020). Though mental health problems can be either causes or consequences of life satisfaction, it is of interest to emphasize that government and educators can improve the level of mental health by improving college students' life satisfaction, such as guiding positive-oriented education about subjective norms, optimism, and active attitudes. Unfortunately, the causal relationships among insomnia, obesity, and mental health were inconclusive in the psychological problem research field (Taylor et al., 2011; Fergusson et al., 2015; Jiang et al., 2021; Sumuer, 2021), which suggested that research using a longitudinal dataset to further investigate the relationships is needed in the future.

There were some limitations that should be noted for this study. Firstly, since the data were collected from a convenience sample of a medical college, the generalizability of the findings to the entire population of general undergraduates should be cautious. Further research has been planned to be conducted in multiple colleges with representative samples. Secondly, due to the cross-sectional survey design, the relationships between mental health and CPD and other variables were not causality. If causality was to be figured out, further studies are needed using a long termly collected data in the future. Thirdly, the degree of CPD and mental health status of interviewees might be different under different pandemic control measures. It would be necessary to conduct a further investigation on the variation of the effects of CPD on mental health when different intervention measures for college students were given. Fourthly, the self-reported variables suffered from recall bias. Some self-reported variables, such as life satisfaction or SWB, could differ by the time of reporting and might be influenced by the individual demographic or socioeconomic characteristics changing over time.

## Conclusions

During the pandemic of COVID-19, college students used mobile phones more frequently, and the duration was nearly 8 h each day on the average. This might be related to the increasing need for learning activities through an online training module and the increasing need for obtaining the latest news and information on the pandemic through mobile phones. The degree of CPD was negatively associated with their mental health status, causing psychological problems, such as anxiety and depression. Thereby, it is necessary to implement policies to guide college students on rationally using cell phones. Engaging in physical exercise and maintaining a good faculty-student relationship could relieve mobile phone addiction. Therefore, it is important to guide and intervene students through more extensive physical training arrangements and more rational assistance to improve the mental status of college students. These findings can help governments and educational policymakers to recognize the impact of the COVID-19 pandemic controlling measures on college students' mental health. Effective interventions are highly needed to prevent severe psychological issues among college students in China during the pandemic.

## Data Availability Statement

The raw data supporting the conclusions of this article will be made available by the authors, without undue reservation.

## Ethics Statement

The studies involving human participants were reviewed and approved by Ethics Committee of Shanghai University of Medicine and Health Sciences. The patients/participants provided their written informed consent to participate in this study.

## Author Contributions

TX and ED made substantial contributions to the study design. TX, XS, and PJ collected data. MC, YY, and ED analyzed the data. TX, XS, and ED interpreted the results of the analysis, completed the manuscripts, and critically revised the manuscript. All authors contributed to the article and approved the submitted version.

## Funding

This work was supported by the National Social Science Foundation of China General Project (Grant No. 19BGL246); the National Social Science Foundation of China Major Project (Grant No. 18ZDA088); and Shanghai 2021 Science and Technology Innovation Action Plan Soft Science Key Project (Grant No. 21692104900). The funders had no role in the question design, analysis, or interpretation.

## Conflict of Interest

The authors declare that the research was conducted in the absence of any commercial or financial relationships that could be construed as a potential conflict of interest.

## Publisher's Note

All claims expressed in this article are solely those of the authors and do not necessarily represent those of their affiliated organizations, or those of the publisher, the editors and the reviewers. Any product that may be evaluated in this article, or claim that may be made by its manufacturer, is not guaranteed or endorsed by the publisher.
