# Supplementary material for: Effects of Cell Phone Dependence on Mental Health Among College Students During the Pandemic of COVID-19: A Cross-Sectional Survey of a Medical University in Shanghai
Source: Front Psychol. 2022 Jun 27;13:920899. doi: 10.3389/fpsyg.2022.920899 (PMC9271901; doi:10.3389/fpsyg.2022.920899)
Supplement: Supplementary file 1 [file Data_Sheet_1.docx]

Appendix 1

**Table S1 The Measurement and Type for Study Variables**

| **Variable** | **How to measure** | **Variable type** |
| --- | --- | --- |
| Gender | Male=1,Female=0 | Categorical |
| Grade | Freshman=1,Sophomore=2,Junior=3,Senior=4 | Categorical |
| Hometown Type | Urban=1,Rural=2 | Categorical |
| Ethnic Group | Han=1,Minority nationality=2 | Categorical |
| Specialty Group | Medical=1, Health economy and management=2 | Categorical |
| Relationship Status | Not dating nor married=1,Dating but unmarried=2,arried=3,；Others=4 | Categorical |
| Monthly Allowances (RMB) | <1000=1,1000-1499=2,1500-1999=3,2000-2499=4,2500-2999=5,>3000=6 | Categorical |
| Insomnia | No=1,Seldom=2,Sometimes=3,Often=4,Daily=5 | Categorical |
| Physical Activity | Never=1,Rare (≤2 times/month)=2,Sometimes (1~2 times/month)=3,Often（3-5 times/month）=4,Daily=5 | Categorical |
| Cigarette Use | Never=1,Ex-smoker=2,Current smoker=3 | Categorical |
| Alcohol Use | Never=1,Rare (≤2 times/month) Sometimes (≤4times/month)=2,Often (≤12 times/month)=3,Always (>12 times/month)=4 | Categorical |
| BMI Index | Low weight=1,Normal=2,Overweight=3,Obesity=4 | Categorical |
| **Phone Use Duration** | Hours/Day | Continuous |
| **Age** | Years | Continuous |
| **DASS** | The total values of 21 items; For each item:Don’t apply to me at all=1, Apply to some degree=2, Apply to me a considerable degree =3,Apply to me very much=4 | Continuous |
| **CPD** | The total values of of 17 items; For each item: not at all =1, Rarely=2, Occasionally=3,Often=4, Always =5 | Continuous |
| **SWLS** | The total values of of 5 items; For each item: Strongly dissatisfied =1,dissatified disa=2, Don’t know=3,satisfied=4, Strongly satisfied =5 | Continuous |
| **SWB** | The total values of of 5 items; For each item: Strongly disagree =1, Disagree=2, Don’t know=3,Agree=4, Strongly agree =5 | Continuous |
| **F-S relationship** | The total values of of 7 items; For each item: Strongly disagree =1, Disagree=2, Don’t know=3,Agree=4, Strongly agree =5 | Continuous |

Appendix 2

**Table S2 Logistic Regression of Sub-dimensions of Depression,Anxiety and Stress on Mental Health of College Students (N=402 )**

| **Variable** | **Depression** | **Anxiaty** | **Stress** |
| --- | --- | --- | --- |
|  | **OR** | **OR** | **OR** |
| **CPD^a^** | 1.080***（1.055-1.106） | 1.071***(1.045 - 1.100) | 1.076*** (1.052 - 1.101) |
| **Cell Phone use Duration^a^** | 1.110***（1.030-1.200） | 1.117***（1.023 - 1.220） | 1.107***（1.030-1.190） |

a:Adjusted for age,gender, F-S relationship, Insomnia, Cigarette use, Physical exercise, BMI, F-S relationship, SWLS,SWB.
